# Albedo and Thermal Ecology of White, Red, and Black Cows (*Bos taurus)* in a Cold Rangeland Environment

**DOI:** 10.3390/ani11051186

**Published:** 2021-04-21

**Authors:** John Derek Scasta

**Affiliations:** Department of Ecosystem Science, University of Wyoming, Laramie, WY 82071, USA; jscasta@uwyo.edu; Tel.: +1-(307)-766-2337

**Keywords:** cold stress, convection, ΔT, heat stress, homoeothermic, solar radiation

## Abstract

**Simple Summary:**

There is a trend of more black hided beef cattle globally, yet cattle on rangelands have limited thermoregulation options. I measured the winter albedo of cows in fresh snow with pixel analysis (*n* = 3 images) and then external cattle temperatures (Temp_cow_), and the differences (ΔT) between Temp_cow_ and ambient air temperature (Temp_amb_) of 638 Temp_cow_ × Temp_amb_ combinations for white (*n* = 183), red (*n* = 158), and black (*n* = 297) *Bos taurus* females. Cattle were free roaming extensive Wyoming, USA rangelands along a broad thermal gradient (≈−33 °C to +33 °C) from 2016 to 2018. Albedo differed for white, red, and black cows (0.69, 0.16, and 0.04, respectively). Temp_cow_ was explained by Temp_amb_, clear sky insolation index, and cow albedo. However, ΔT was explained by Temp_amb_, long-wave infrared radiation, Temp_cow_, and cow albedo. Temp_cow_ suggests red and black cows experience ~2× higher values than white cows at the coldest temperatures.

**Abstract:**

Cattle in high-elevation rangelands experience cold and hot extremes. Given the increase in black hided cattle globally, thermoregulation options on rangelands, and hide color function affecting mammal thermal ecology, this study quantified winter albedo, external cattle temperatures (Temp_cow_), and differences (ΔT) between Temp_cow_ and ambient air temperature (Temp_amb_), for different color cattle along a thermal gradient (≈−33 °C to +33 °C). From 2016 to 2018, I measured 638 individual Temp_cow_ × Temp_amb_ combinations for white (*n* = 183), red (*n* = 158), and black (*n* = 297) *Bos taurus* female cattle free roaming extensive Wyoming, USA rangelands. Pixel brightness of cow images relative to snow indicated mean (±standard error) albedo for white, red, and black cows (*n* = 3 of each) was 0.69 (±0.15), 0.16 (±0.04), and 0.04 (±0.01), respectively (*p* = 0.0027). Temp_cow_ was explained by Temp_amb_ (+), clear sky insolation index (+), and cow albedo (−). However, ΔT was explained by Temp_amb_ (−), long-wave radiation (infrared; Rad_LW_ (−)), Temp_cow_ (+), and cow albedo (+). Temp_amb_ relative to ΔT was correlated for all hide colors (all *p*-values < 0.0001; all *r*^2^ values > 0.7)), yet slopes (*m*) were ~2× greater for red and black cows than white cows.

## 1. Introduction

Thermal stress on livestock has implications for animal performance, animal welfare, and in extreme cases, even animal survival. Such stressful thermal situations can occur at both the warm and cold ends of the thermal gradient. Given recent climate change and warming trends globally [1], much attention has been given to heat stress and the well documented effects of heat stress on livestock, which include reduced feed intake, reduced performance, and death in extreme cases [2,3]. Given the forecasted rise in global temperature, much of the research has been appropriately focused on heat stress; however, cold stress can be equally problematic as it affects a large proportion of global livestock production regions at discrete periods of the year and induces hormonal and adaptive changes [4]. In North America alone, nearly 2/3 of all livestock inhabit regions with subzero mean temperatures in the winter season (below 0 °C; [4]).

As temperatures decrease and cold stress escalates, animal physiology acclimates to adapt to the lower temperatures with consequences for animal function and production. When external temperatures reach a critically low threshold for livestock, animals move out of the zone of thermoneutrality and into a cold thermogenesis stage where metabolism adapts to generate body heat as a coping mechanism (i.e., elevated basal metabolic intensity) [5]. The consequence of this metabolic cold adaptation is an increase in feed requirements ranging from 30 to 70% in beef cattle [6]. Given the seasonal variation of hot and cold conditions, maintenance energy requirements of cattle also fluctuate drastically in northern latitudes and/or high altitudes during periods of forage deficiencies with realized reductions in animal performance as determined by feed-to-gain ratios [4]. These animal issues are largely a product of the environment that animals inhabit and the associated climatic patterns.

Consequently, environments that experience large thermal gradients across seasons and extreme events present challenging livestock production environments. Environments such as cold steppe high-elevation rangelands often occur near the interface of plains and mountains, in mountainous regions, or in arid deserts and may experience drastic diurnal and seasonal temperature variation. Experimental approaches to understand how animals adapt to such variable thermal environments have included experimental approaches where animals were exposed to cold (3 °C), thermoneutral (25 °C), or hot (35 °C) conditions in artificially housed environments [7], or where animals were confined with limited ability to use the landscape in order to optimally thermoregulate [2]. 

Thermoregulation is fundamental to physiological and distributional adaptations of thermal conditions for all organisms. Mammals, such as cattle, are homeothermic due to their constant body temperature that is regulated relative to the ambient environmental temperature. Relatedly, the surface temperature of the mammal has an important role in radiative balance and energy loss. It is therefore imperative to understand the difference between the surface temperature of an organism and the temperature of the surrounding environment [8] which is affected by the interface with phenotypic traits such as hide color. Calculation of the difference between the surface temperature of an organism and the temperature of the surrounding environment (ΔT) is fundamental to animal thermal ecology because ΔT is necessary to calculate forced convection, which is heat transfer between an organism and its environment (Equation (1); [8]),

Forced convection (Q) = h_c_AΔT (1)

where h_c_ is the surface heat transfer coefficient and A is the surface area of the body of interest. Here, the critical role of an organism’s boundary layer thickness and wind speed is accounted for in the calculation of h_c_ (Equation (2); [8]),

Surface heat transfer coefficient (h_c_) = f(V^k^_1_/D^k^_2_) (2)

where h_c_ is a function of both the dimension of the organism, D, and wind speed, V, where the exponents k represent combinations of thermal conductivity, thermal capacity, and thermal expansion coefficients (for which experimental determination for an organism is noted to be technically demanding and not often performed [8]). Similarly, ΔT is also important for calculating net radiation (Q_n_), which is the difference between radiation emitted and radiation received, considered as net loss of energy as heat (Equation (3); [8]),

Net radiation (Q_n_) = Aεσ(T_o_^4^ − T_e_^4^) (3)

where ε is emissivity, σ is the Stefan–Boltzmann constant, T_o_ is the organism’s temperature, and T_e_ is the environmental temperature. 

However, thermal stress of animals is not simply a function of the ambient temperature, but also the interaction with solar radiation, a consideration that was largely neglected in early efforts to develop guidelines for minimizing thermal stress during the transportation phase of livestock production systems [3]. This radiant energy emitted by the sun can vary due to a range of environmental conditions such as cloudiness [9] and approximately half is within the short-wave portion of the electromagnetic spectrum (i.e., visible) while the remainder is within both the infrared and ultraviolet portions [10]. 

Surface properties of an organism, in particular phenotypically expressed hide color, influence the proportion of solar radiation that is reflected. Functionally, this is experienced by an organism when darker surfaces feel hotter and lighter surfaces feel cooler—a concept referred to as albedo [9]. Albedo affects the amount of radiant energy absorbed or reflected, and is a unitless measure that ranges from 0 to 1, where 0 reflects no solar radiation and 1 reflects all of the solar radiation. Albedo is important for remote sensing and energy balance calculations [11]. Generally, black surfaces are closer to 0 because they absorb the majority of the solar radiation (feel hotter) and white surfaces are closer to 1 because they reflect the majority of the solar radiation (feel cooler). 

Consequently, animal hide color and albedo have implications for thermal ecology and thermal stress mitigation. From a practical standpoint, thermal stress mitigation attempts to manipulate the heat load balance where reducing heat loss is the aim during cold stress, but reducing heat load and/or increasing heat loss are the aim during heat stress [3]. For extensive systems where reducing heat load is not possible, such as the case for free-roaming animals in rangeland environments, developing basic thermal ecology information to develop more thermal stress-tolerant animals may be the only viable alternative. The other options for reducing thermal stress identified in a decision tree analysis include (1) relocating to a more suitable area, (2) modifying the environment in the same location, (3) changing operations, or (4) stop producing livestock—all of which are impractical for livestock production enterprises generally [3]. Thus, the notion of matching the animal to the environment has been suggested to be a primary strategy for adapting to environmental variation and extremes and understanding the role of animal albedo is a part of that process [12].

Considering albedo and thermal stress-tolerant animals, it is also important to understand trends in livestock selection and national herd composition. In many regions of the world, the proportion of black beef cattle has been increasing and recent reports indicate that ~75% of all US beef cows were black in 2012 [13,14]. Therefore, given the trend towards more black hided cattle in the US domestic beef herd, that high-elevation rangelands can experience both cold and hot thermal extremes, and the limitation of existing research that has primarily examined cattle in artificial thermal environments that negate solar radiation or only examined cattle in confined feeding operations, I sought to quantify the external surface temperatures of cattle, the difference between the surface temperature of an organism and the temperature of the surrounding environment (ΔT), and albedo estimates for black, red, and white cattle along a broad thermal gradient. 

## 2. Materials and Methods

### 2.1. Study Site

The study area is a high-elevation rangeland approximately 2190 m a.s.l. near Laramie, WY, USA. This region receives 250–360 cm of precipitation annually and has a mean monthly temperature range from 0.1 to 26.7 °C. The study area is classified as *BSk* (*B*—Arid; *S*—Steppe; *k*—cold) according to the Köppen–Geiger climate classification due to it being temperate, continental, with winter snow, and having a broad thermal gradient [15]. This open rangeland environment inherently provides little to no shade due to its short vegetation and thus, animals are fully exposed to solar radiation, which is not uncommon in global rangelands used for livestock grazing [16]. Cows were provided by the University of Wyoming (UW) Beef Unit and consisted of approximately 40 white Charolais, 250 black Angus, and 40 red Angus and/or Hereford. Cows were multiparous, estimated to be 3 to 7 years of age, and were sampled across a range of lactation and gestation stages that occur across winter and summer seasons. Cattle management followed the guidelines stated in the Guide for the Care and Use of Agricultural Animals in Research and Training [17] and the UW Institutional Animal Care and Use Committee (IACUC Protocol #20170508DS0060-01). 

### 2.2. Cow Albedo Calculations and Analysis

**Figure 1:**
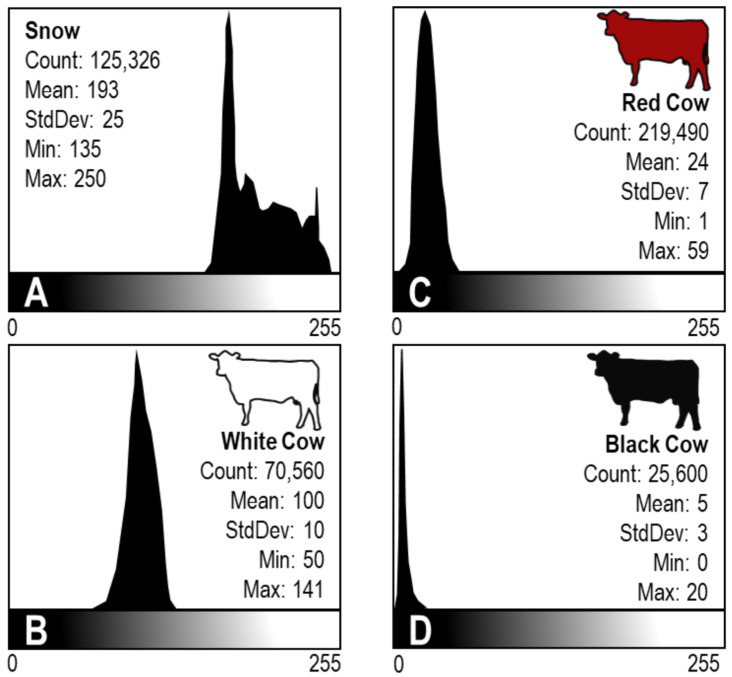
Example of image analysis histograms of frequency of occurrences of different gray pixel values as an indicator of brightness. Each histogram represents pixels selected for (**A**) snow, (**B**) a white cow, (**C**) a red cow, and (**D**) a black cow, concurrently in a photo. Photos were taken in January 2016 west of Laramie, WY, USA at the University of Wyoming, Agricultural Experiment Station, Laramie Research and Extension Center—Beef Unit and cattle were *Bos taurus* cows. Analyses were conducted using open-source image analysis software ImageJ.

On 6 January 2017, I captured 3 digital images of commingled white, red, and black *Bos taurus* cows in the snow using an 8-megapixel camera and then used the open-source image analysis software ImageJ to calculate winter albedo values for the different cow colors (Figure 1; [18]). Cows at the time of sampling were in the 2nd trimester of gestation and non-lactating. 

In each image, snow pixels in the image were analyzed as the reference and considered the baseline constant using a typical snow albedo value of 0.85 [19,20]. For each image, I developed a histogram of the frequency of occurrences of different gray values of selected snow and different colored cow pixels (Figure 1). The mean of these gray value occurrences is an indicator of the brightness of selected pixels which is possible because digital images are produced by a combination of red, green, and blue primary color pixels and each color has a brightness value ranging from 0 to 255 (Figure 1). I used three different images as replicates and then calculated the mean albedo value for snow, and white, red, and black cows within each image [21,22]. For each cow color, I first calculated the relative albedo which is a cow:snow brightness ratio (Albedo_rel_; Equation (4) [22]),

Albedo_rel_ = Bright_cow_ ÷ Bright_snow_ (4)

where Bright_cow_ is the mean brightness of specific cow hide pixels (i.e., black, red, or white cows) and Bright_snow_ is the mean brightness of snow pixels which functions as reference pixels. I then calculated absolute albedo for each cow color (Albedo_abs_; Equation (5)),

Albedo_abs_ = Albedo_rel_ × 0.85 (5)

where Albedo_rel_ is multiplied by the snow albedo reference value of 0.85 [11,19,22].

Albedo values range from 0 to 1 and were therefore transformed with an arcsine transformation. Data were assessed for normality with a Shapiro–Wilk test and W-statistics and *p*-values indicated a deviation from normality. Thus, I then used a non-parametric Kruskal–Wallis test with hide color as a fixed effect. Finally, with defined alternative hypotheses (HA) for multiple paired comparisons (specifically, HA1: white cow albedo > red cow albedo, HA2: red cow albedo > black cow albedo, and HA3: white cow albedo > black cow albedo), I calculated effect sizes using Cohen’s *d* to understand the magnitude of differences. 

### 2.3. Cow Thermal Measurements and Relating Temp_amb_ to Temp_cow_

**Figure 2:**
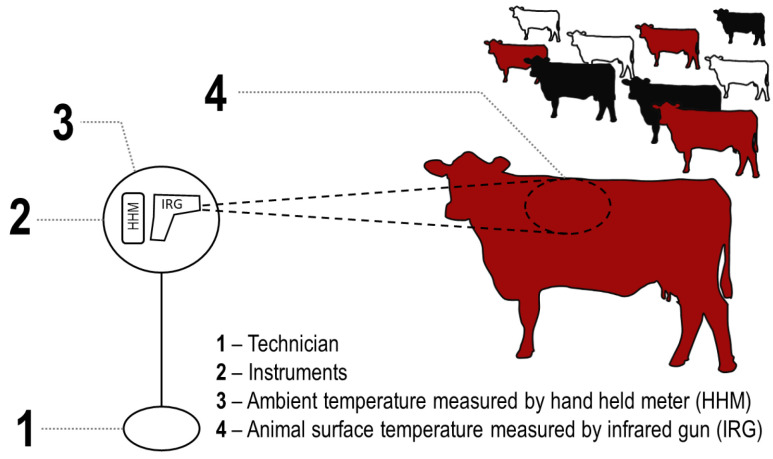
Field method for in situ measurement of ambient environmental temperature (Temp_amb_) and cattle surface temperature (Temp_cow_) using two separate thermal instruments simultaneously operated by a single technician. Temp_amb_ measured with a handheld meter (HHM; Kestrel^®^ 3000). Temp_cow_ measured with an infrared gun (IRG) high temperature thermometer (Extech Instruments^®^ 42,545 made by FLIR Systems^®^) with the laser placed approximately at the top of the shoulder from a distance of 5 to 15 m and sensed temperature was recorded. Temp_amb_ and Temp_cow_ were recorded simultaneously with HHM and IRG instruments in the same physical position. Cattle were white, red, and black *Bos taurus* cows free roaming on rangeland west of Laramie, WY, USA at the University of Wyoming, Agricultural Experiment Station, Laramie Research and Extension Center—Beef Unit.

**Figure 3:**
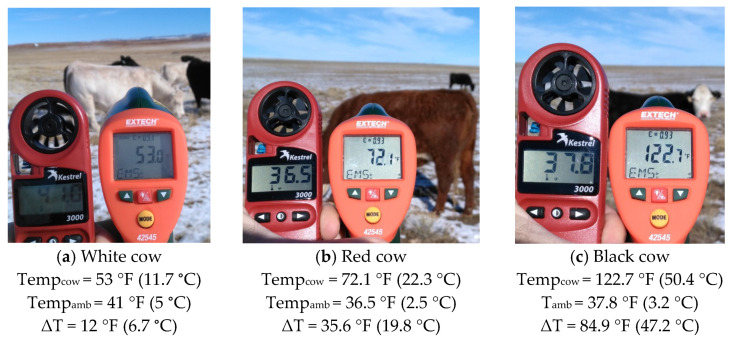
Examples of in situ measurement of ambient environmental temperature (Temp_amb_) and cattle surface temperature (Temp_cow_) using a handheld meter (HHM; Kestrel^®^ 3000) and an infrared gun (IRG) high temperature thermometer (Extech Instruments^®^ 42,545 made by FLIR Systems^®^). Temperatures collected from animals and the environment in situ were then used to calculate the difference (ΔT) between Temp_cow_ and Temp_amb_. Cattle were white, red, and black *Bos taurus* cows free roaming on rangeland west of Laramie, WY, USA at the University of Wyoming, Agriculture Experiment Station, Laramie Research and Extension Center—Beef Unit.

From 2016 to 2018, I measured 638 individual cattle surface temperature × ambient environmental temperature combinations for the same *Bos taurus* female cow groups as described above (*n*_white_ = 183, *n*_red_ = 158, *n*_black_ = 297). At any discrete sampling interval, I was able to collect at least 14 measurements per hide color group. I only used female cattle because change in rectal temperatures of cattle relative to handling may be influenced by sex [23]. This study quantified cattle surface temperature (Temp_cow_) and ambient environmental temperature (Temp_amb_) using two separate thermal instruments simultaneously operated (Figure 2). For Temp_cow_, I used an infrared (IR) high temperature thermometer (Extech Instruments^®^ 42,545 made by FLIR Systems^®^, Wilsonville, OR, USA) sensor with a temperature range of −50 to 1000 °C, a distance to target ratio of 50:1, and 0.93 emissivity setting (base setting). The sensor was placed approximately at the top of the shoulder from a distance of 5 to 15 m and sensed temperature was recorded. For Temp_amb_, I used a handheld weather meter (Kestrel^®^ 3000, Boothwyn, PA, USA) that was in the same physical position as the IR sensor and recorded Temp_amb_ at the same approximate time as Temp_cow_ (Figure 3). All temperatures were measured in degrees Fahrenheit and converted to degrees Celsius (Figure 3). I then calculated the absolute difference (ΔT) between Temp_cow_ and Temp_amb_.

### 2.4. Weather and Solar Radiation Data 

In addition to the in situ derived data described above for cow albedo, Temp_cow_, and Temp_amb_, I acquired additional weather and solar radiation data from remotely sensed and publicly available sources based on *a priori* justification for inclusion in cow thermal ecology modeling as described in greater detail in Table 1. First, I acquired local weather data from the station located at the Laramie Regional Airport west of Laramie, WY, USA, which borders the research ranch, for daily average humidity, wind speed, and dewpoint temperature. Second, I acquired daily maximum vapor pressure deficit (VPD) estimates from the Parameter-elevation Relationships on Independent Slopes Model (PRISM) interpolation method that extrapolates data based on a digital elevation model (DEM) at a resolution of 2.5 arcmin (~4-km). PRISM data are derived from some 10,000 stations weighted relative to physiographic similarity to a grid cell of interest [24,25]. I used the cell at latitude 41.2990 and longitude −105.6540 with data from the AN81d dataset. Third, I used the same coordinates for the PRISM data to acquire daily solar radiation data from National Aeronautics and Space Administration (NASA) satellites through the Prediction Of Worldwide Energy Resources (POWER) project for earth skin temperature (Temp_earth_), clear sky insolation index (KTclear), long-wave radiation (infrared; Rad_LW_), and short-wave radiation (visible; Rad_SW_) which is produced natively on a global 1° × 1° latitude/longitude grid and then remapped to a 0.5° × 0.5° latitude/longitude grid scale via bilinear interpolation and/or replication [26]. 

### 2.5. Temp_cow_ and ΔT Modeling

I conducted a model selection analysis using Akaike’s information criterion corrected for small sample (AICc) sizes first for Temp_cow_ and then separately for ΔT using the AICcmodavg package in R [38,39]. Prior to modeling, I first assessed correlation coefficients for explanatory variables acquired from similar sources (in situ animal or environmental measurement, local weather station measurement, PRISM-extrapolated measurement, or NASA satellite-derived measurement) to exclude similarly sourced autocorrelated explanatory variables from final models using a cutoff of *r* > 0.7 [40,41]. Consequently, Temp_earth_ was highly correlated with both Rad_LW_ and Rad_SW_ variables and thus removed, humidity was highly correlated with wind speed and thus removed, and VPD was highly correlated with Temp_dew_ and thus removed. 

I then conducted each model selection analysis in categorical steps. Step one was to determine the top “weather” variable and included Temp_amb_, Temp_dew_, and humidity. Step two was to determine the top “solar radiation” variable and included Rad_LW_, Rad_SW_, and KTClear. Step three was to then build top candidate models that included the top models from the first modeling steps with the additional incorporation of in situ animal measurements [42]. Null models were included in each step. This approach allows for the identification of the most informative yet most parsimonious model explaining a response variable. At each categorical step, I scaled AICc values to the top model by relativizing the model with the lowest AICc (i.e., the top model in the step) set at zero and then calculating differences between the top model and each of the other models (ΔAICc) in order to rank models. Models that had ΔAICc scores ≤ 2 were considered to be in the top set of models. Akaike weights (ω_i_) indicate the relative likelihood of models and were used to determine top models [42]. I then calculated 95% confidence intervals in a final screening approach to identify uninformative variables in final models [42].

Finally, I also used linear least squares regression to analyze how Temp_amb_ predicts ΔT as stratified by cow hide color. Strength of relationships was determined based on the correlation coefficient (*r*^2^) and significant statistical differences based on α = 0.05. To compare the slopes of the regression lines as stratified by cow hide color, I then used an analysis of covariance (ANCOVA) with Tukey’s corrected *p*-value as an indicator of significant differences in a post hoc comparison. Due to unequal variances as indicated by a Levene’s test, I also included a Kruskal–Wallis test. Finally, due to missing identification tags and cows being culled/added over time, I was unable to track individual animals and thus, no repeated measures were accounted for in any analyses.

## 3. Results

The pixel analysis histograms revealed different frequency of occurrences of gray values for snow, white cows, red cows, and black cows along the dark to bright gradient (Figure 1A–D). In the example provided from the analyses of one of the images in Figure 1A–D, such differences are apparent. For snow pixels, the mean value was 193 which occurs on the bright end of the scale, with a range from 135 to 250 (Figure 1A). Snow pixels had a standard deviation (SD) of 25 which indicates greater variation than of the cows (white cow SD = 10, red cow SD = 7, and black cow SD = 3), which is likely due to shadows or other microspatial features on the surface (Figure 1A–D). Likewise, the low variation for black cows is likely due to a lack of confounding effects from shadows. For white cow pixels, the mean value was 100 which occurs near the middle of the scale, with a range from 50 to 141 (Figure 1B). For red cows, the mean value was 24 which occurs near the dark end of the scale, with a range from 1 to 59 (Figure 1C). For black cows, the mean value was 5 which occurs nearly at the darkest end of the scale with a range from 0 to 20 (Figure 1D). 

**Figure 4:**
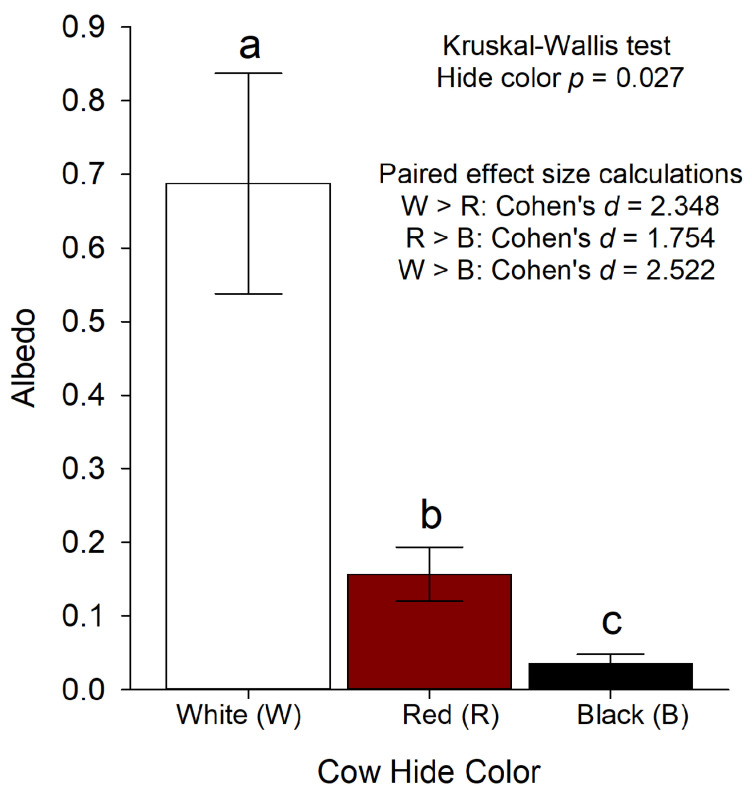
Winter albedo values for white, red, and black cows. Albedo values derived from pixel analyses with snow albedo as a reference. Data were transformed with an arcsine transformation and then analyzed with a non-parametric Kruskal–Wallis test with hide color as the fixed effect. Cohen’s *d* effect sizes were calculated to understand the magnitude of differences based on the defined alternative hypotheses (HA) for each paired comparison; specifically, HA1: white cow albedo > red cow albedo, HA2: red cow albedo > black cow albedo, and HA3: white cow albedo > black cow albedo. Significant statistical differences were based on α = 0.05 (and indicated by different letters; (**a**–**c**)). Cattle were red, white, and black *Bos taurus* cows free roaming on rangeland west of Laramie, WY, USA at the University of Wyoming, Agriculture Experiment Station, Laramie Research and Extension Center—Beef Unit.

Cow winter albedo values differed significantly relative to cow hide color (*p*-value = 0.027; Figure 4). Mean white cow albedo = 0.69 (±0.15 standard error (SE)), mean red cow albedo = 0.16 (±0.04 SE), and mean black cow albedo = 0.04 (±0.1 SE) (Figure 4). The effect of the differences was greatest between white and black cows (Cohen’s d = 2.522) and the least between red and black cows (Cohen’s d = 1.754) (Figure 4). 

For the in situ and satellite-derived measurements, means and ranges are presented in Table 2. Mean Temp_amb_ was 4.4 °C with a range from −32.8 to 35.6 °C. Mean Temp_cow_ was 32.4 °C with a range from −10.7 to 63.1 °C. ΔT mean was 28.0 °C with a range from −2.2 to 83.3 °C. When stratified by hide color, ΔT mean for white cows was 48.7 °C with a range of −2.2–48.7 °C, ΔT mean for red cows was 70.8 °C with a range from −1.8 to 70.8 °C, and ΔT mean for black cows was 83.3 °C with a range from −0.9 to 83.3 °C. Weather data in this study included Temp_dew_ with a mean of −10.0 °C and a range from −27.6 to 7.1 °C, humidity with a mean of 56.7% and a range from 9.0 to 84.0%, VPD with a mean of 13.5 hPa and a range from 0.6 to 32.5 hPa, and wind speed with a mean of 29.0 km/h and a range from 11.3 to 53.1 km/h. Satellite-derived measurements in this study included KTClear with a mean of 0.6 and a range from 0.3 to 0.8, Temp_earth_ with a mean of −0.1 °C and a range from −24.6 to 23.0 °C, Rad_LW_ with a mean of 5.9 kW hr per m^2^ per day and a range from 3.6 to 8.7 kW hr per m^2^ per day, and Rad_SW_ with a mean of 3.9 kW hr per m^2^ per day and a range from 1.3 to 8.5 kW hr per m^2^ per day. 

For the first information theory modeling approach with Temp_cow_ as the response variable, the top weather model was Temp_amb_ and neither Temp_dew_ nor wind speed were competitive (Table 3). Akaike weights (ω_i_) suggest Temp_amb_ was orders of magnitude more informative for Temp_cow_ than Temp_dew_ or wind speed. The top solar radiation model was KTClear and neither Rad_LW_ nor Rad_SW_ were competitive (Table 3). Akaike weights suggest KTClear was 97 times more informative for Temp_cow_ than Rad_LW_ or Rad_SW_. The overall top model included Temp_amb_, KTClear, and Albedo and was orders of magnitude more informative for Temp_cow_ than any other model. The parameter estimate for Temp_amb_ was positive indicating that as ambient environmental temperatures increase, so does Temp_cow_. The parameter estimate for KTClear was also positive, indicating that as the proportion of total solar radiation that reaches the surface of the earth increases, so does Temp_cow_. The parameter estimate for Albedo was negative, indicating an inverse relationship where lower albedo results in higher Temp_cow_. The 95% confidence intervals for these three parameter estimates all overlap zero and indicate that Temp_amb_, KTClear, and Albedo regulate Temp_cow_ and are informative explanatory variables (Table 4).

For the second information theory modeling approach with ΔT as the response variable, the top weather model was Temp_amb_ and neither Temp_dew_ nor wind speed were competitive (Table 5). Akaike weights suggest Temp_amb_ was orders of magnitude more informative for ΔT than Temp_dew_ or wind speed. The top solar radiation model was Rad_LW_ and neither Rad_SW_ nor KTClear were competitive (Table 5). Akaike weights suggest Rad_LW_ was orders of magnitude more informative for ΔT than Rad_SW_ or KTClear. The overall top model included Temp_amb_, Rad_LW_, Albedo, and Temp_cow_ and was orders of magnitude more informative for ΔT than any other model. The parameter estimate for Temp_amb_ was negative, indicating that as ambient environmental temperatures increase, ΔT decreases. The parameter estimate for Rad_LW_ was also negative, indicating that as long-wave solar radiation (infrared) increases, ΔT decreases. The parameter estimate for Albedo was negative, indicating an inverse relationship where lower albedo results in ΔT. The parameter estimate for Temp_cow_ was positive, indicating that as the external cow surface temperatures increase then so does ΔT. The 95% confidence intervals for these four parameter estimates all overlap zero and indicate that Temp_amb_, Rad_LW_, Albedo, and Temp_cow_ regulate ΔT and are informative explanatory variables (Table 6). 

**Figure 5:**
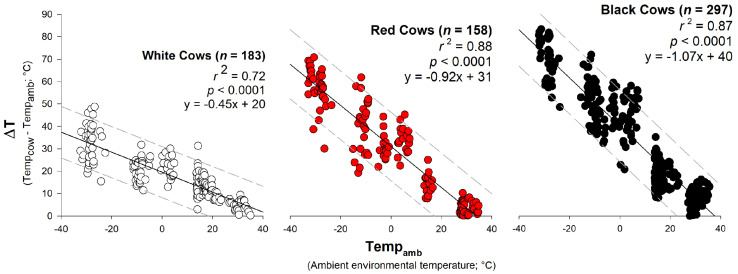
Linear least-squares regression to analyze how ambient environmental temperature (Temp_amb_) predicts ΔT (the difference between cattle surface temperature (Temp_cow_) and Temp_amb_) stratified by cow hide color for white, red, and black cows. Strength of relationships was determined based on the correlation coefficient (*r*^2^) and significant statistical differences based on α = 0.05. Cattle were red, white, and black *Bos taurus* cows free roaming on rangeland west of Laramie, WY, USA at the University of Wyoming, Agriculture Experiment Station, Laramie Research and Extension Center—Beef Unit. Dashed lines represent 95% prediction bands.

Temp_amb_ was highly corelated (all *r*^2^ values > 0.7) and a significant predictor of ΔT for all cow hide colors (all *p*-values < 0.0001) (Figure 5). The slopes (m) of the relationships were also significantly different according to ANCOVA and Kruskal–Wallis post hoc comparisons (all *p*-values < 0.001) suggesting different ΔT responses; for example, for white cows m = −0.45, for red cows m = −0.92, and for black cows m = −1.07, which indicate this relationship is ~2× stronger for red and black cows than for white cows. In addition, the negative m values indicate that ΔT decreases as temperatures increase (Figure 5). 

## 4. Discussion

This experiment has established unique winter albedo reference values concomitantly for white, red, and black *Bos taurus* cows for the first time in the published literature [11]. These reference values are important for examining the thermal ecology of phenotypically diverse cattle, particularly in the context of the external surface temperatures of cows relative to solar radiation drivers in both the infrared and visible spectrums [9]. The variation of winter albedo values within a single hide color in this study suggests that assessment of genotypic-driven stress tolerance should include interactions with phenotypic-driven variation in albedo, even within a uniform colored group of cattle [43]. Moreover, in many production environments, including the environment of this study, the landscape is very open with little to no shade available to cattle [16]. An important caveat to this study is the albedo values were derived during the middle of winter when pixel brightness of digital images could be relativized to snow in the image—a scenario that is not possible in the summer. This context must be emphasized because during the winter, cows have hair coats that tend to be thicker and rougher than the thinner and sleeker coat of the summer. This is important because evidence suggests that the summer coat may have a higher albedo value which can help decrease the solar heat load [44]. 

Looking to the future, the preference for black hided cattle and subsequent increase in such cattle in the US and other regions [13,14], coupled with climatic change that includes increasing temperatures [1], should lead to more focus on these interactions. For example, these results suggest that black cattle may suffer more thermal stress (“heat stress”) in the summer while white cattle may suffer more thermal stress (“cold stress”) in the winter. Furthermore, this may also suggest that such contrasting advantages and disadvantages may have implications for selecting black cattle for colder high-altitude and high-latitude environments and white cattle for warmer tropical environments, particularly considering the climate extremes on opposite ends of the thermal gradient in such contrasting environments. Moreover, understanding the variation of performance of heterogeneous groups of cows [45] or growing cattle such as yearling steers or heifers [46,47], in harsh environments, may be enhanced by understanding cow albedo and thermal ecology. Ultimately, the understanding of solar radiation × hide color interactions has implications for optimizing cattle selection and breeding for thermoregulatory advantages in different environments. 

The differential role of various solar metrics for predicting Temp_cow_ versus ΔT also provides greater insight for how cattle actually experience temperature [8]. While ambient temperature is relevant for applied livestock management questions or ecological insights, it is the combination of ambient temperature, radiation, albedo, and heat transfer that collectively influence operative temperature [48]. Operative temperature is considered to be the “thermal stress placed on an organism with specified external properties” [49]. Thus, scientists should therefore move beyond only measuring external and internal animal temperatures and propel forward to relating those temperatures to ambient temperatures relative to the external properties of the animal with the understanding that certain radiation metrics are more influential than others. In this study, the role of long-wave radiation was most informative for ΔT, likely due to how visible short-wave radiation penetrates into the coat but how infrared long-wave radiation is absorbed at the surface of the coat [34]. 

The variable winter albedo and ΔT values relative to different cow hide color groups may also have implications for cold stress which can have negative consequences for animal production [6]. As temperatures drop and cold stress escalates, animals move out of the zone of thermoneutrality as they pass lower critical temperature and enter a cold thermogenesis stage where cold stress manifests and animals alter metabolic rate to generate heat and cope with the cold [5]. At the coldest temperatures, cows with the highest albedo values (white cows) had the lowest ΔT values, whereas cows with the lowest albedo values (black cows) had the highest ΔT values—values that were nearly 2× higher. Future research needs to further assess if such an effect translates into different metabolic rates and energy requirements. Based on the role of ΔT in the calculation of forced convection which is heat transfer between an organism and its environment [8], logic suggests that black cattle would have greater forced convection rates than white cattle. Additional measurements to understand variation of boundary layer depth and density (i.e., animal hair depth and density) and subsequent interactions with wind speed and Temp_amb_ will also help to enhance the understanding of heat transfer of cattle. Moreover, additional data on the relative clearness or cloudiness of a region and the confounding factor of cloud cover should also be integrated into future studies [28].

The minimal role of wind speed for predicting either Temp_cow_ or ΔT in this study is also notable because wind speed is known to be critically important in surface temperature change as it compresses and reduces the animal boundary layer as it increases [36]. In other words, the convection coefficient is known to be inversely related to the boundary layer thickness (i.e., animal hair depth) which is compressed as wind speed increases [36]. More specifically, the calculation of the surface heat transfer coefficient (h_c_) includes wind speed [8]. The results reported herein, in this context of wind speed, indicate that at the scales measured, and in the context of other variables, wind speed was not as important for predicting either animal temperature response variable, as other solar, temperature, or albedo variables—particularly across the broad cold to hot gradient assessed. 

## 5. Conclusions

Quantifying albedo of commingled cattle of different hide colors is basic scientific progress for applied livestock research. As genetic selection for certain genotypes and phenotypes oscillates through time, some phenotypes become more abundant such as the current trend for black hided cattle in the US [13,14]. Such industry trends, when coupled with climatic trends, facilitate thermal ecological interactions between animals and their environment that have implications for animal welfare and animal production. For example, there may be thermoregulatory advantages for cows of different color hide colors in different seasons or different regions due to solar radiation × hide color interactions—black or red hide cattle in cold periods or cold regions and white hide cattle in warm periods or warm regions. Globally though, there are latitudinal geographic zones that differ in solar energy and thermal stress and the temperate zones of both hemispheres are places where cattle may experience both cold stress in the winter and heat stress in the summer. Understanding how external coat characteristics of animals influence how they experience temperature, particularly when temperatures drop below the zone of thermoneutrality and animals enter cold thermogenesis [5], is essential to developing novel strategies for cold stress mitigation in these temperate zones of the world. While such information has application for domestic beef cattle, it also leads to the need to better understand the evolutionary adaptations of wild animals such as American bison (*Bison bison*) which span broad thermal gradients and are dark colored, and polar bears (*Ursus maritimus*) which occur in very open and cold environments and are white. In addition to quantifying cow albedo, the understanding that different satellite-derived clearness and radiation metrics have differential influences on variable thermal metrics of cattle is important to guide researchers to measure the most appropriate drivers of thermal stress [34]. 

## Figures and Tables

**Table 1:** Potential predictor variables and associated justification for inclusion for modeling external surface temperatures of red, white, and black hided cattle (Temp_cow_) and then the difference (ΔT) between Temp_cow_ and the ambient temperature of the surrounding environment (Temp_amb_). Variables are listed in alphabetical order. Cattle were white, red, and black *Bos taurus* cows free roaming on rangeland west of Laramie, WY, USA at the University of Wyoming, Agriculture Experiment Station—Beef Unit.

Variable	Biological Rationale and Justification for Inclusion in Modeling
Ambient environmental temperature (Temp_amb_) ^a^	Also noted as air temperature, this is important to determine an organism’s deviation from the zone of thermoneutrality and influences convection coefficients [27].
Clear sky insolation index (KTClear) ^d^	Used for the weather prediction of intra-day solar forecasting [28] and for daylight utilization in farm animal production [29]. Fraction of insolation available at the top of the atmosphere and an indication of the total solar radiation incident on a horizontal earth surface.
Cow winter albedo ^1,a^	Commonly used in remote sensing research, this refers to the proportion of solar radiation reflected by any surface (water, land, organismal), where darker surfaces reflect less than lighter surfaces and is important for determining energy balance [30]. Measured in winter when snow was available to relativize pixel brightness in images.
Cow surface temperature (Temp_cow_) ^a^	The surface temperature of an organism can be used to predict convective heat loss [27].
Dewpoint temperature (Temp_dew_) ^c^	The temperature to which air must be cooled, relative to pressure and water-vapor content, in order to reach saturation and for dew or condensation to form; this influences evaporative heat loss [31].
Earth skin temperature (Temp_earth_) ^d^	The earth’s surface temperature as opposed to the meteorological definition of surface temperature which is actually measured by suspended air thermometers above the surface of the earth; this reflects the thermal environments of physiologically diverse organisms [32].
Humidity ^b^	The amount of water vapor that is in the atmosphere; humidity interacts with temperature and influences thermal stress of animals [33]. Humidity is also important given the temperature–humidity index (THI) which can have a combined interactive impact [3].
Long-wave radiation (infrared; Rad_LW_) ^d^	Downward thermal infrared long-wave radiative flux ^d^; has an important role in predicting radiative heat gain for animals with different hide colors, particularly at the surface of the animal [34]; may govern simulated temperatures of wildlife species [35].
Short-wave radiation (visible; Rad_SW_) ^d^	^d^All sky insolation incident on a horizontal surface (short-wave) ^d^; short-wave solar radiation has been used to develop a thermal stress index for dairy cows in Brazil [9].
Vapor pressure deficit (VPD) ^c^	The difference between the amount of moisture in the air and the maximum moisture the air can hold at saturation; this affects the exchange of the energy between an animal and its environment through an interaction with temperature [36,37].
Wind speed ^b^	Also noted as wind flow speed, this is a basic atmospheric measurement of air moving from high to low pressure often driven by temperature flux. This variable is considered in the calculation of the convection coefficient and an influential for predicting the surface temperature of an organism [36].

^1^ Determined through analysis of digital pixels with cows in fresh snow and relativized with published snow albedo values; ^a–d^ Data Source (^a^ Field Measurement, ^b^ Laramie Airport Weather Station, ^c^ PRISM [25], ^d^ NASA [26]).

**Table 2:** Units, means, minimums, and maximums of potential predictor variables and associated justification for inclusion for modeling external surface temperatures of red, white, and black hided cattle (Temp_cow_) and then the difference (ΔT) between Temp_cow_ and the ambient temperature of the surrounding environment (Temp_amb_). Variables are listed in alphabetical order. Cattle were white, red, and black *Bos taurus* cows free roaming on rangeland west of Laramie, WY, USA at the University of Wyoming, Agriculture Experiment Station, Laramie Research and Extension Center—Beef Unit.

Variable	Unit	Mean	Minimum	Maximum
Ambient environmental temperature (Temp_amb_)	°C	4.4	−32.8	35.6
Clear sky insolation index (KTClear)	unitless index; 0–1	0.6	0.3	0.8
Cow winter albedo	unitless index; 0–255	0.25	0.04 (black cows)	0.69 (white cows)
Cow surface temperature (Temp_cow_)	°C	32.4	−10.7	63.1
Dewpoint temperature (Temp_dew_)	°C	−10.0	−27.6	7.1
Earth skin temperature (Temp_earth_)	°C	−0.1	−24.6	23.0
Humidity	%	56.7	9.0	84.0
Long-wave radiation (infrared; Rad_LW_)	kW hr per m^2^ per day	5.9	3.6	8.7
Short-wave radiation (visible; Rad_SW_)	kW hr per m^2^ per day	3.9	1.3	8.5
Vapor pressure deficit (VPD maximum)	hPa	13.5	0.6	32.5
Wind speed	km/h	29.0	11.3	53.1

**Table 3:** Weather, radiation, and cow albedo models assessed for cow surface temperature (Temp_cow_) for white, red, and black *Bos taurus* cows near Laramie, WY, USA.

Model	K	AICc	ΔAICc	ω_i_
Step 1—Weather Models				
Ambient Temperature (Temp_amb_)	3	4967.08	0.00	1
Dewpoint Temperature (Temp_dew_)	3	4980.38	13.30	0
Wind Speed	3	5023.33	56.25	0
Null	2	5023.93	56.85	0
Step 2—Radiation Models				
Clear Sky Insolation (KTClear)	3	4988.16	0.00	0.97
Long-wave Radiation (Rad_LW_)	3	4995.78	7.62	0.02
Short-wave Radiation (Rad_SW_)	3	4998.08	9.92	0.01
Null	2	5023.93	35.77	0.00
Step 3—Top Models + Winter Albedo				
Temp_amb_ + KTClear + Albedo	5	4674.57	0.00	1
Temp_amb_ + KTClear	4	4959.79	285.22	0
Temp_amb_	3	4967.08	292.51	0
KTClear	3	4988.16	313.58	0
Null	2	5023.93	349.35	0

**Table 4:** Top candidate model parameter estimates and 95% confidence intervals for cow surface temperature (Temp_cow_) for white, red, and black *Bos taurus* cows near Laramie, WY, USA.

Variable	Estimate	Lower	Upper
Ambient Temperature (Temp_amb_)	0.1770	0.1328	0.2213
Clear Sky Insolation (KTClear)	19.172	13.1404	25.2043
Albedo	−27.026	−29.8800	−24.1729

**Table 5:** Weather, radiation, and cow albedo models assessed for the difference (ΔT) between external surface temperatures of cattle (Temp_cow_) and the ambient temperature of the surrounding environment (Temp_amb_) for white, red, and black *Bos taurus* cows near Laramie, WY, USA.

Model	K	AICc	Delta AICc	Weight
Step 1—Weather Models				
Ambient Temperature (Temp_amb_)	3	4967.08	0.00	1
Dewpoint Temperature (Temp_dew_)	3	5184.00	216.92	0
Wind Speed	3	5540.83	573.75	0
Null	2	5684.51	717.43	0
Step 2—Radiation Models				
Long-wave Radiation (Rad_LW_)	3	5142.32	0.00	1
Short-wave Radiation (Rad_SW_)	3	5258.25	115.93	0
Clear Sky Insolation (KTClear)	3	5616.83	474.51	0
Null	2	5684.51	542.19	0
Step 3—Top Models + Animal Attributes [Winter Albedo and Temp_cow_]				
Temp_amb_ + Rad_LW_ + Albedo + Temp_cow_	6	−38,308.95	0.00	1
Temp_amb_ + Rad_LW_ + Temp_cow_	5	−38,197.42	111.53	0
Temp_amb_ + Rad_LW_ + Albedo	5	4674.41	42,983.36	0
Temp_amb_ + Rad_LW_	4	4961.41	43,270.42	0
Rad_LW_	3	5142.32	43,451.27	0
Temp_amb_	3	4967.08	43,276.03	0
Null	2	5684.51	43,993.46	0

**Table 6:** Top candidate model parameter estimates and 95% confidence intervals for the difference (ΔT) between external surface temperatures of cattle (Temp_cow_) and the ambient temperature of the surrounding environment (Temp_amb_) for white, red, and black *Bos taurus* cows near Laramie, WY, USA.

Variable	Estimate	Lower	Upper
Ambient Temperature (Temp_amb_)	−0.8230	−0.8672	−0.7787
Long-wave radiation (Rad_LW_)	−8.3971	−8.9595	−7.8347
Albedo	−24.160	−29.8022	−18.5184
Cow Surface Temperature (Temp_cow_)	0.5022	0.37772	0.62661

## Data Availability

Data can be made available upon request.
